# Helix Capping in RNA Structure

**DOI:** 10.1371/journal.pone.0093664

**Published:** 2014-04-01

**Authors:** Jung C. Lee, Robin R. Gutell

**Affiliations:** 1 BioMolecular Engineering Program, Physics and Chemistry Department, Milwaukee School of Engineering, Milwaukee, Wisconsin, United States of America; 2 Center for Computational Biology and Bioinformatics, Institute for Cellular and Molecular Biology, and Section of Integrative Biology, the University of Texas at Austin, Austin, Texas, United States of America; Dundee University, United Kingdom

## Abstract

Helices are an essential element in defining the three-dimensional architecture of structured RNAs. While internal basepairs in a canonical helix stack on both sides, the ends of the helix stack on only one side and are exposed to the loop side, thus susceptible to fraying unless they are protected. While coaxial stacking has long been known to stabilize helix ends by directly stacking two canonical helices coaxially, based on analysis of helix-loop junctions in RNA crystal structures, herein we describe *helix capping*, topological stacking of a helix end with a basepair or an unpaired nucleotide from the loop side, which in turn protects helix ends. Beyond the topological protection of helix ends against fraying, helix capping should confer greater stability onto the resulting *composite helices*. Our analysis also reveals that this general motif is associated with the formation of tertiary structure interactions. Greater knowledge about the dynamics at the helix-junctions in the secondary structure should enhance the prediction of RNA secondary structure with a richer set of energetic rules and help better understand the folding of a secondary structure into its three-dimensional structure. These together suggest that helix capping likely play a fundamental role in driving RNA folding.

## Introduction

RNA is an active participant in the chemistry of life. While mRNAs code for proteins, other RNAs including structured RNAs are responsible for many essential cellular processes, ranging from protein synthesis to gene expression and regulation [1], [2], [3], [4]. Structured RNAs fold hierarchically from their sequence into their native, three-dimensional tertiary structure [5], [6], [7], [8]. While the computational determination of RNA tertiary structure is still beyond our reach, bioinformatic comparative sequence analysis accurately predicted the secondary structures of various structured RNAs [9], composed of a large number of very short canonical helices and loops that are rearranged into its native tertiary structure, mostly with the help of metal ions such as Mg^2+^ and Na^+^ [10]. While RNA folding has been explored from different perspectives, the helix-loop junctions in the secondary structure can potentially have a significant influence on the prediction of higher-order RNA structure and long-range tertiary interactions since the energetics of helices can potentially be improved with knowledge about the junctions.

Together with basepairing interactions, base-stacking contributes significantly to the stability of DNA and RNA helices [11], [12], [13], [14], [15], [16], [17], [18], [19]. While internal basepairs are stacked on both sides, the ends of RNA secondary structure helices are stacked on their internal side and exposed to the loop side, potentially susceptible to fraying in that their imino protons exchange with solvent [20], [21], [22]. Thus, short RNA helices can potentially unfold as fraying can propagate from the ends of helices towards the interior. How do short canonical helices prevent their unfolding prior to their assembly into its three-dimensional structure? The ends of short canonical helices in structured RNAs, however, are frequently flanked by tetraloops [23], lonepair triloops [24], G:A and A:A basepairs [25], or other canonical helices [26]. Consistently, previous melting studies have shown that canonical RNA helices are greatly stabilized in the presence of tetraloops or various mismatches at their ends [13], [17], [27], [28], [29], [30], [31], [32]. In particular, UUCG and GAAA tetraloops are known to nucleate the formation of unusually stable hairpin structures and serve as a reverse transcription termination signal of bacteriophage T4 mRNA or as a rho-independent transcription terminator of prokaryotic mRNAs [27], [33]. This example suggest that other recurrent structural elements or motifs can protect and stabilize the ends of short canonical RNA helices against fraying, reminiscent of α-helix capping in protein [34], [35], [36].

Nonetheless no systematic analysis of the helix-loop junctions in large naturally occurring structured RNAs has been documented to address the protection of helix ends from fraying. Based on our detailed and comprehensive analysis of the helix-loop junctions in the high-resolution *Thermus thermophilus* 16S rRNA (T16S) and *Haloarcula marismortui* 23S rRNA (H23S) crystal structures [37], [38], herein we explore *helix capping motifs*, single basepairs or unpaired nucleotides capable of protecting the ends of canonical RNA helices (**see Materials and Methods for definition**).

## Materials and Methods

A canonical RNA helix is defined as an antiparallel A-form RNA duplex with at least two consecutive basepairs, each forming a canonical (standard Watson-Crick or wobble) conformation regardless of its basepair group [39]. The RNA helices in the crystal structures were visually examined how helix ends are potentially protected from the loop side. While coaxial positioning of two canonical helices is called *coaxial stacking*. topological stacking of a helix end with a capping motif – a basepair or an unpaired nucleotide from the loop – is termed *helix capping* if the vertical distance from the helix end to the capping motif is similar to the one (∼3.0 Å) between two consecutive internal basepairs in a canonical helix. Various RNAs, including the 16S and 23S rRNAs from the *Thermus thermophiles* 30S (T16S) and *Haloarcula marismortui* 50S (H23S) crystal structures [37], [38].

## Results

### Identification of short canonical RNA helices and their topological end-stacking

**Figure 1:**
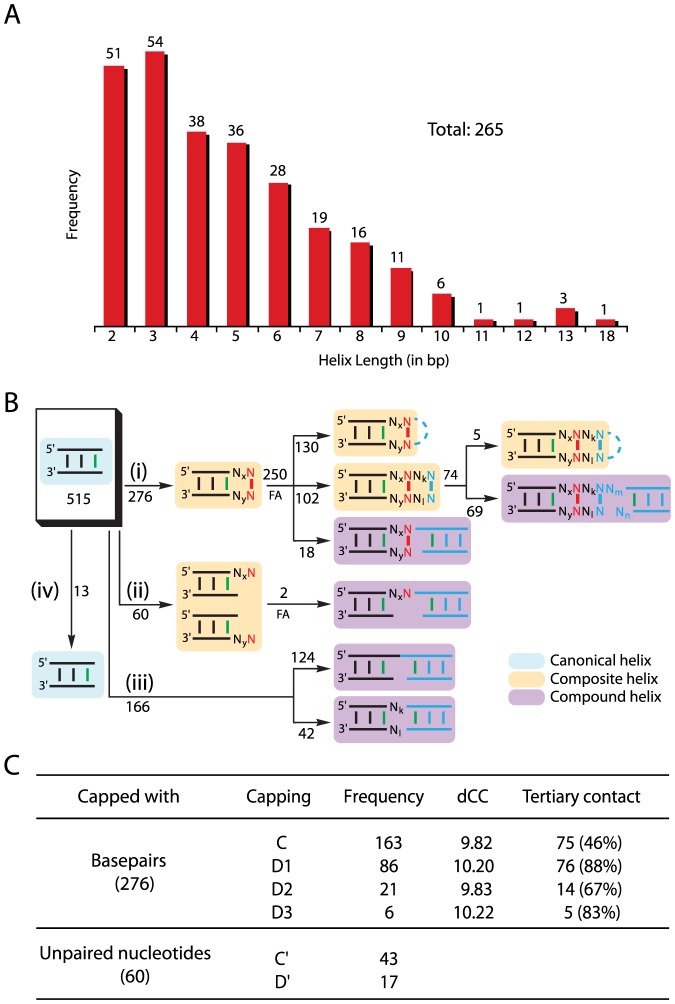
C’nonical helices found in T16S and H23S rRNAs and their end-stacking. (**A**) Distribution of canonical helix lengths. (**B**) Schematic representation of topological end-stacking of canonical helices, where *x* and *y* refer to the numbers of the intervening nucleotides between a canonical helix and a helix capping motif. While helix ends are shown in green and helix capping motifs in red, the 3′- and 5′-IVS are denoted with N_*x*_ and N_*y*_, respectively, where N = {A, C, G, U}. (**i**) Capping with basepairs: C-capping if *x* = 0 and *y* = 0; D1-capping if *x* = 0 and *y*>0; D2-capping if *x*>0 and *y* = 0; D3-capping if *x*>0 and *y*>0. (**ii**) Capping with unpaired nucleotides: C′-capping if *x* = 0 or *y* = 0; D′-capping if *x*>0 or *y*>0. (**iii**) Coaxial stacking without any “bridging” cap. (**iv**) Canonical helices not involved in any type of end-stacking. Further association (FA) to the loop side with additional unpaired nucleotides, basepairs, and other canonical helices are shown in cyan dotted lines, cyan basepairs, and cyan helices, respectively. Each of *x, y, k, l, m,* and *n* is any integer greater than or equal to zero. (**C**) Distribution of helix capping motifs and their involvement in tertiary contact.

While, *a priori*, we expect longer helices to be enthalpically more stable than shorter helices, our analysis revealed that the vast majority of the 265 canonical RNA helices identified in T16S and H23S are very short, with the median length of 4 bp, compared to a complete helical turn of 11–12 bp for the A-form RNA (**Figure 1A**). Analysis of the helix-loop junctions of these canonical helices surprisingly revealed that all but 13 (97%) of the 515 resolved helix ends are topologically involved in end-stacking from the loop side (**Figure 1B**). Specifically, while 166 ends are involved exclusively in coaxial stacking of two canonical helices to form a *compound helix*, 336 are capped, 276 with basepairs and 60 with unpaired nucleotides, forming a *composite helix* or bridging two canonical helices stack coaxially. Besides, these identified helix capping motifs are frequently involved in long-range tertiary contacts (**Figure 1C**). Additional analysis demonstrated that nearly all helix ends in other classes of structured RNAs are involved in end-stacking (**Table S1**). Provided that helix ends fray [20], [21], [22], such preponderance of end-stacking in structured RNAs reflects its significance not merely in protecting short canonical RNA helices against fraying as indicated by earlier studies [12], [13], [14], [15], [16], [17], [18], [19], [27], [28], [29], [30], [31], [32].

### Topological classification of helix capping motifs

**Figure 2:**
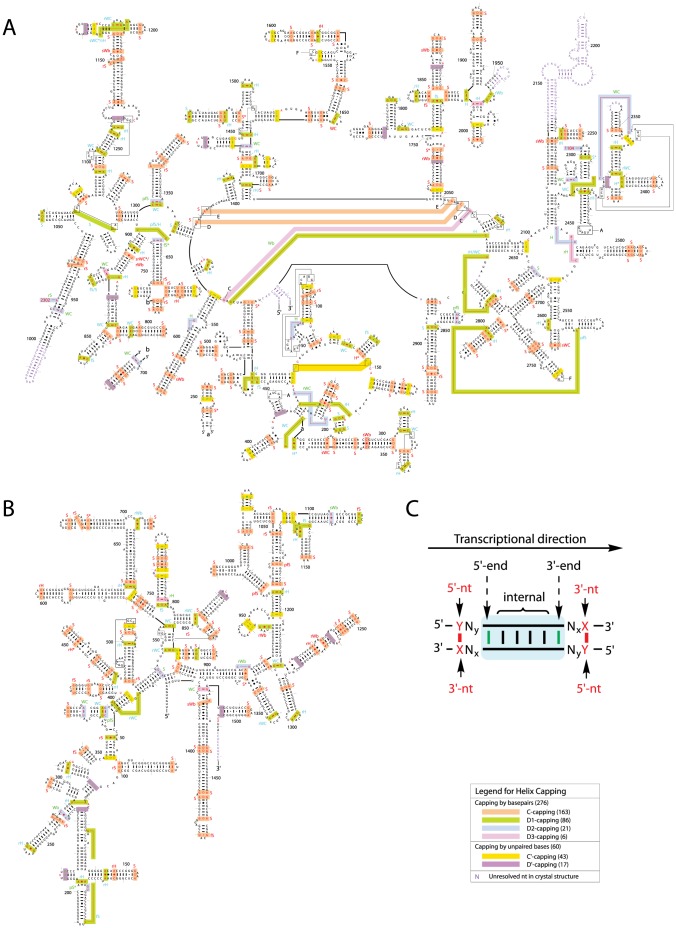
Helix capping motifs mapped onto the rRNA secondary structures. (**A and B**) Identified helix capping motifs in H23S and T16S, respectively. While a helix end capped with a basepair is represented by the capping basepair (red), a helix end capped with an unpaired nucleotide is represented by the helix end (green). The tick marks are for the nucleotide numbers in the H. marismortui 23S and T. thermophilus 16S rRNA; (C) Schematic representation of both helix ends (5′-end and 3′-end) and the 3′- and 5′-nt in a helix capping basepair (X:Y), where X, Y = {A, C, G, U}.

A detailed analysis of the 336 basepairs and unpaired nucleotides that cap helix ends, or helix capping motifs (**Figure 2**), revealed that helix capping occurs contiguously (*C-capping* with basepairs and *C′-capping* with unpaired nucleotides) or discontiguously (*D-capping* with basepairs and *D′-capping* with unpaired nucleotides), depending on the absence or presence of the intervening sequence of nucleotides (IVS) between a canonical helix end and its helix capping motif, respectively (**Figure 1B**). The arrangement of the IVS is 5′ or/and 3′ to a canonical helix further distinguishes D-capping into D1, D2, and D3. The IVS can be either short (1–3 nt) or long (∼25 to 1000 nt); if short, the bases of the IVS are usually flipped out of a composite helix, making tertiary contacts implicated in RNA folding (**see below**).

Overall, C-capping occurs more frequently than D-capping (163 vs. 113), and C′-capping occurs >2-fold more frequently than D′-capping (43 vs. 17) (**Figure 1C**). Interestingly, while any of the ten basepair groups [39] can serve as a capping basepair motif, all but 21 adopt non-canonical conformations with varying C1′-C1′ distances (dCC's); the exceptional 21 form the canonical conformations (**Table 1**). In addition, any of the four nucleotides (A, C, G, and U) can be an unpaired capping nucleotide (**Table 2**). Nonetheless, helix capping motifs are largely biased for a few basepair groups or unpaired nucleotides, depending on the types of helix capping.

**Table 1:** Diversity of capping basepairs and their conformations (bpC)† and dCC.

Capping basepairs (X:Y)		(3′, 5′)‡	bpC(frequency)	dCC (Å)&
**C-caps**	**163**	**(11, 133)**		**9.820**
G:A	100	(0, 100)	S(90) rS(9) H(1)	9.517
C:A	16	(3, 13)	S(7) rH(3) pfS(2) sWb(2) rS(1) rWb(1)	10.042
U:C	10	(1, 9)	S(6) sWC(2) rS(1) rWC(1)	10.271
A:A	9	―	S(4) rS(3) rH(1) rWb(1)	10.235
U:G	9	(6, 3)	S(6) fS(3)	10.617
U:A	8	(0, 8)	S(6) rH(1) pfS(1)	10.121
C:C	6	―	sWb(4) S(2)	10.029
G:G	4	―	S(4)	11.001
C:G	1	(1, 0)	rS(1)	13.745
**D1-caps**	**86**	**(7, 64)**		**10.198**
U:A	37	(1, 36)	rH(27) rWC(5) WC(3) sWC(1) H(1)	9.911
C:G	12	(1, 11)	WC(10) fS(1) rWC(1)	10.589
G:A	12	(2, 10)	S(8) fS(2) pfS(1) H(1)	9.577
A:A	8	―	rH(3) S(1) fS(1) rS(1) pfS(1) rWb(1)	11.005
C:A	5	(2, 3)	rH(3) fS(1) H(1)	10.461
U:G	5	(1, 4)	fS(2) S(1) rWb(1) pS(1)	10.578
G:G	4	―	rH(4)	11.703
C:C	2	―	S(1) rH(1)	9.632
U:U	1	―	rWb(1)	9.008
**D2-caps**	**21**	**(6, 10)**		**9.829**
C:G	6	(1, 5)	WC(4) pfS(2)	11.164
U:A	5	(4, 1)	WC(2) H(1) fS(1) rWC(1)	9.641
G:A	3	(0, 3)	S(2), H(1)	10.260
A:A	2	―	fS(1) rS(1)	10.012
U:G	1	(1, 0)	pS(1)	6.813
C:A	1	(0, 1)	rWb(1)	11.390
G:G	1	―	H(1)	11.831
C:C	1	―	Wb(1)	7.785
U:U	1	―	sWb(1)	8.516
**D3-caps**	**6**	**(1, 5)**		**10.222**
C:G	2	(0, 2)	WC(2)	10.390
U:A	2	(0, 2)	rH(2)	9.555
C:A	1	(1, 0)	rH(1)	11.100
U:G	1	(0, 1)	Wb(1)	10.347

† Capping basepairs and their conformations are according to the Lee-Gutell system [39]: WC, Watson-Crick; Wb, wobble; S, sheared; rS, reversed sheared; fS, flipped sheared; pfS, parallel flipped sheared; H, Hoosteen; rH, reversed Hoogsteen; sWC, slipped Watson-Crick; sWb, slipped wobble; rWC, reversed Watson-Crick; rWb, reversed wobble; pS, parallel sheared.

‡ (3′, 5′) refers to the frequency of hetero capping basepair motifs (X:Y) whose Y is topologically located immediately 3′ and 5′ to a canonical helix, respectively.

& The average dCC's for the canonical basepairs in the A-form RNA in T16S and H23S are 10.599 Å and 10.563 Å, respectively.

**Table 2:** Diversity of unpaired capping nucleotides.

Capping nucleotides	C′-caps (3′, 5′)†	D′-caps	Total
A	23 (17, 6)	9	32
C	2 (2, 0)	3	5
G	13 (12, 1)	2	15
U	5 (5, 0)	3	8
Total	43 (36, 7)	17	60
	31 (27, 4)‡		

† (3′, 5′) refers to the frequency of unpaired capping nucleotides that are immediately 3′ and 5′ to a canonical helix, respectively.

‡ 31 helix ends immediately flanked by two unpaired capping nucleotides, one at 3′ and the other at 5′.

C-capping occurs predominantly with G:A (100; 61%), followed by C:A (16: 10%), with the sheared conformation occurring most frequently (125; 77%) (**Table 1**), consistent with our previous study showing that helix ends in rRNA comparative structure models are frequently juxtaposed with highly conserved G:A and A:A baspairs [25]. D1-capping occurs most commonly with U:A (37; 43%), followed by C:G (12; 14%) and G:A (12; 14%), with the reversed Hoogsteen conformation occurring most frequently(38; 44%); C:G and G:A D1-caps dominantly adopt the Watson-Crick (10; 83%) and sheared (8; 67%) conformations, respectively. The infrequent D2- and D3-cappings involve C:G and U:A most frequently but adopt various conformations. Overall, the C:A C-caps and the A:A D1-caps are most diverse in their conformations, each adopting six different conformations. In contrast, G:G C- and D1-caps invariably form the sheared and reversed Hoogsteen conformations, respectively. Interestingly, all but 25 (or 89%) of the 237 hetero capping basepairs have the Y at the 5′-nt position (**Table 1**). Helix capping unpaired nucleotides are most frequently A (32; 53%), followed by G (15; 25%), and the vast majority (36; 84%) of the 43 C′-caps are actually a 3′-dangling nucleotide (**Table 2**).

### Helix capping versus helix stability

**Figure 3:**
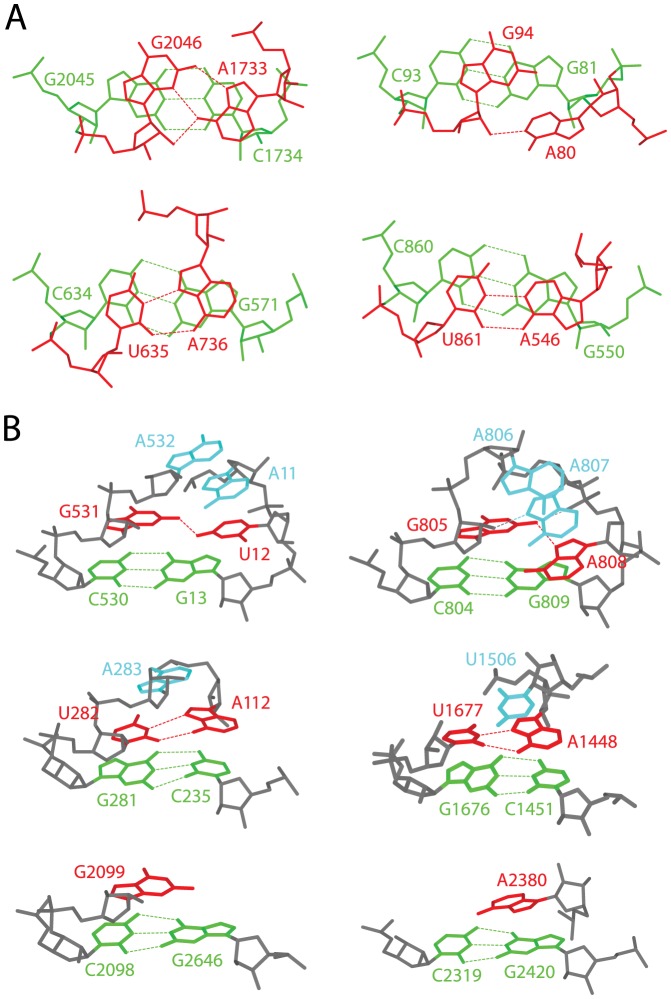
Representative helix capping motifs in rRNAs and their topological protection of helix ends against fraying. (**A**) Stacking patterns of capping basepair motifs over helix ends: G:A C-caps (upper panels) in the sheared (G2046:A1733 in H23S, left) and reversed sheared (G94:A80 in H23S, right) conformation; U:A D-caps (lower panels) in the reversed Hoogsteen (U635:A736 in T16S, left) and reversed Watson-Crick (U861:A546 in T16S, right) conformation. (**B**) Helix capping motifs further associated with forming hairpin-like loop folds over helix ends: A C-cap in a multistem loop, U12:G531 in H23S (top left), is further involved in forming a structure similar to the GNRA tetraloop (top right); D-caps in multistem loops, A112:U282 in T16S and A1448:U1677 in H23S, are further involved in forming an overall hairpin-like loop fold (middle panels); Unpaired capping nucleotides, G2099 C′-cap and A2380 D′-cap in H23S, form a hairpin-like structure by themselves (bottom panels). As in Figure 1, while helix ends are shown in green and helix capping motifs in red, the nucleotides further associated from the loop side are shown in cyan.

While both nucleotides in all helix capping basepairs, except for 13 C-caps, stack well on top of a helix end by predominantly forming a non-canonical conformation, all the helix capping unpaired nucleotides stack right on top of the hydrogen-bonding interface of a helix end (**Figure 3**). A detailed basepair stacking analysis in canonical RNA helices revealed that one base of a basepair stacks up on top of its immediately 5′ flanking basepair while the other base only marginally stacks on the 5′ flanking basepair. This indicates that helix capping motifs overall stack better on a helix end than an internal basepair does in a canonical helix. The exceptional 13 C-caps (9 G:A's, 3 A:A's, and 1 C:A), all in the reversed sheared conformation [39], overall assume a hairpin-like loop of a single nucleotide, similar to that observed with helix capping unpaired nucleotide motifs; with the 3′-nt stacked directly on top of the hydrogen-bonding interface of a helix end, the 5′-nt gets displaced into the minor groove (**Figure 3A** **, upper right**). These together strongly suggest that helix capping motifs stabilize short canonical helices by restricting the fraying entropy at helix ends.

Of the 336 helix capping motifs, a total of 252 (or 75%) are part of either a larger RNA structural motif that has been previously described [23], [24], [40], [41], [42], [43], [44], [45] or their mimics, some mediating coaxial stacking between two flanking canonical helices (**Figures 1B** **and** **3B**). Given that canonical RNA helices are dramatically stabilized by the presence of UNCG and GNRA tetraloops [27], [28], [29], [30], these additional associations of a helix capping motif are likely to provide additional stabilization to a composite helix that is already stabilized by the helix capping motif itself.

### Tertiary contacts formed around helix capping motifs and their role in RNA folding

**Figure 4:**
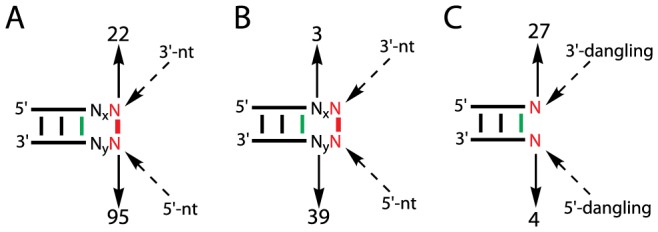
Preferred tertiary contact sites around helix capping basepair motifs and favored C′-capping. (**A**) Tertiary contacts favoring the 5′-nt over the 3′-nt; (**B**) Tertiary contacts favoring the 5′-IVS over the 3′-IVS; (**C**) C′-capping favoring 3′ to canonical helices. While N = {A, C, G, U}, x and y are any integer greater than or equal to zero. As in Figure 1, while helix ends are in green, helix capping motifs are in red.

Helix capping basepair motifs and their associated IVS frequently participate in tertiary contacts, contributing to the folding of the RNA secondary structure into its three-dimensional structure. Overall, while less than a half (75; 46%) of the 163 C-caps form tertiary contacts, the vast majority (95; 84%) of the 113 D-caps and their associated IVS participate in tertiary interactions (**Figure 1C**). In particular, 30 of the 95 D-caps involved in tertiary contacts are by themselves long-range tertiary basepairs, each bringing two remote regions on the secondary structure into contact, having initiated the transition from the secondary to the tertiary structure. Surprisingly, the tertiary contacts formed by helix capping basepair motifs occur far more frequently through the 5′-nt than through the 3′-nt (95 vs. 22) (**Figure 4A**). A further analysis revealed that the). 5′-nt A in G:A C-caps is the primary site for long-range tertiary contacts in all but one GNRA tetraloops found in T16S and H23S; with the 3′-nt G in the G:A C-caps stacked right on top of the basepairing interface of a helix end, the 5′-nt A is slightly displaced toward the minor groove and forms a single hydrogen-bond from its N7 to the G NH_2_, leaving its N1 and N3 available for tertiary contacts (**Figure 3B** **, top right**). More surprisingly, the tertiary contacts made by the IVS associated with D-caps occur almost exclusively through the 5′-IVS (**Figure 4B**). Furthermore, when two unpaired nucleotides are simultaneously available immediately 3′ and 5′ to a helix end, C′-capping is favored 7-fold with the one 3′ to the helix end over the one 5′ to the helix end (27 vs. 4) (**Table 1** and **Figure 4C**), consistent with previous melting studies demonstrating that a 3′-dangling nucleotide stabilize a canonical helix far more than a 5′-dangling nucleotide does [12], [13], [14], [15], [16], [19]. Altogether, these suggest that, while stabilizing helix ends against fraying, the 5′-nt of helix capping basepair motifs and its associated IVS be rather intrinsically entropic, making many long-range tertiary contacts largely responsible for hierarchically driving RNA folding.

### Dependence of helix capping on basepair polarities at helix ends

An analysis of the 336 capped helix ends revealed that, while helix capping favors the 3′-end only marginally over the 5′-end, the overall frequency order for the capped helix ends is C:G>G:C>U:G>U:A>A:U>G:U, with two-thirds accounted for by the most frequent C:G and G:C ends (**Table S2**). This strongly suggested a correlation between helix-ending basepair identity and helix capping frequency, prompting us to further elucidate the dependence of helix capping frequency on the basepair polarities of the two terminal basepairs at helix ends in T16S and H23S.

This additional analysis revealed that, while overall helix ends favor Y:R (297; 58%) over R:Y (171; 33%), the Y:R ends are more than twice more likely to be capped than the R:Y ends (208 vs. 93) (**Table 3**). With the basepair polarities of the last two terminal basepairs combined, the helix ends with the Y:R|Y:R polarity are capped most frequently (75%), those with the Y:R|R:Y polarity least frequently (42%) and the remaining two in between (66%), strikingly consistent with the NMR melting temperatures of self-complementary tetramers, 5′-GGCC-3′ (54.0°C)>5′-GCGC-3′ (49.9°C)>5′-CCGG-3′ (47.8°C)>5′-CGCG-3′ (36.9°C) [46]. This reflects that helix capping strongly favors energetically more stable but short canonical helices, stabilizing the growing number of short canonical RNA helices being formed early in RNA folding.

**Table 3:** Polarities of the last two terminal basepairs and their effect on helix capping.

Terminal basepairs†	All helix ends‡	Capped helix ends only‡							
		C	D1	D2	D3	C′	D′	Total	%capped
**5′-YY**	139	55	26	6	2	10	5	104	75%
**3′-RR**									
**5′-YR**	64	9	7	2	0	6	3	27	42%
**3′-RY**									
**5′-RY**	158	54	24	6	2	11	7	104	66%
**3′-YR**									
**5′-RR**	107	31	21	5	1	12	1	71	66%
**3′-YY**									
**Other**	47	14	8	2	1	4	1	30	64%
**Total**	515	163	86	21	6	43	17	336	65%

† Polarities of the last two terminal basepairs at helix ends, where Y:R = {C:G, U:A, U:G} and R:Y = {G:C, A:U, G:U}.

‡ Data were from an analysis of the 515 helix ends resolved in T16S and H23S.

### Conformational diversity of helix capping basepair motifs

While helix capping basepair motifs can be any of the 10 basepair groups in different conformations [39], they are strongly biased for a few basepair groups and conformations, depending on the types of helix capping (**Table 1**). C-caps are most frequently G:A, followed by C:A, adopting predominantly the sheared conformation. D1-caps are biased toward U:A, C:G, and G:A, forming dominantly the reversed Hoogsteen, Watson-Crick, and sheared conformations, respectively. Both D2- and D3-caps are most commonly C:G and U:A, forming frequently the Watson-Crick conformation. In particular, the majority of the non-canonical conformations adopted by helix capping basepair motifs has a significantly shorter dCC compared to 10.6 Å in a canonical basepair in the A-form RNA, topologically effectively protecting helix ends against fraying. An additional analysis revealed that 84 (or 30%) of the 276 capping basepairs are involved in RNA-protein interactions (unpublished data). Nonetheless, only 10 of them could change their conformation in the presence of protein, suggesting that the conformational diversity of capping basepairs will not be biased by the presence of protein.

A few of the helix capping basepairs including C:A and U:A form several different conformations, albeit with identical or very similar sequence and structural contexts, demonstrating that they are susceptible to structural perturbation from the entropic loop side and may undergo dynamic conformational changes as RNA folds into its native tertiary structure. An analysis of the archaeal *H. marismortui* and bacterial *E. coli* 23S rRNA crystal structures [38], [47] revealed five homologous helix capping basepairs whose conformations are completely different in the two crystal structures (**Table 4**). In particular, the two, including H23S-0873∶0876 and H23S-1164∶1192, share exactly the same sequence and structural context between the two phylogenetically distant organisms, strongly supporting the idea of dynamic conformational changes but without affecting the overall RNA structure and function.

**Table 4:** Capping basepairs in different conformations in different crystal structures†.

Positions of capping basepairs	H23S (1S72)	E23S (2QAM)
H23S-0644∶0903 (E23S-0587∶0810)	G:U Wb	C:U H
H23S-0768∶0893 (E23S-0677∶0800)	U:C srWC*	A:A rWb
H23S-0873∶0876 (E23S-0780∶0783)	G:A fS	G:A S
H23S-1164∶1192 (E23S-1060∶1088)	U:A sWC*	U:A rH
H23S-2661∶2812 (E23S-2626∶2777)	U:A rH	C:G WC

† Data are from an analysis of the 23S rRNA crystal structures of *H. marismortui* (H23S0) [38] and *E. coli* (E23S) [47],and the basepair conformations are based on the Lee-Gutell system [39].

## Discussion

Our ability to predict RNA secondary and tertiary structure is mostly dependent on our detailed understanding of many different structural motifs and the organizing principle explaining how they are assembled to form the complex, but highly ordered three-dimensional tertiary structure. Given that the vast majority (96%) of helix ends in structured RNAs are either capped (65%) or coaxially stacked (31%) from the loop side (**Table S1**), both helix capping and coaxial stacking play roles in defining RNA structure and driving RNA folding. In particular, helix capping not only locks and stabilizes the fraying ends of many short canonical helices formed early in RNA folding, but facilitate the formation of many long-range tertiary contacts that are, in cooperation with coaxial stacking, essential for defining the complex three-dimensional architecture of structured RNAs. Besides, helix capping in RNA favors intrinsically more stable helix ends, working cooperatively with the sequence polarity of the last two terminal basepairs to drive helix formation during RNA folding. Thus, the derivation of the stabilizing energies of all the identified helix capping motifs and their subsequent application to the development of an RNA folding algorithm would greatly enhance our capability of predicting RNA secondary and tertiary structure.

Such data for mismatches (C-caps) and dangling nucleotides (C′-caps) have been derived calorimetrically [14], [15], [16], [17], [18], [19] and employed in the energy-based *mfold* RNA folding program [48]. Nonetheless, not all calorimetric data for the identified helix capping motifs are currently available, especially for those implicated in folding the secondary into the tertiary structure. Due to the complexity of experimental design, however, it is presently experimentally challenging to obtain the stabilizing energies for the D- and D′-caps. An alternative is to compute their evolutionary frequency enriched in a set of homologous RNA sequences from a wide range of different organisms, followed by employing them as a proxy for their experimental energy. In addition, the determination and implementation of polarity-dependent nearest-neighbor energies for the last two terminal basepairs at helix ends could further improve the accuracy of RNA structure prediction from sequence.

## Supporting Information

Table S1 **End-stacking of canonical helices in various RNA crystal structures.**
(EPS)

Table S2 **Identity of helix-ending basepairs and its influence on helix capping direction.**
(EPS)
